# Clinical Characteristics-Assisted Risk Stratification for Extent of Thyroidectomy in Patients With 1–4 cm Solitary Intrathyroidal Differentiated Thyroid Cancer

**DOI:** 10.3389/fendo.2021.790730

**Published:** 2022-02-08

**Authors:** Fang Dong, Lin Zhou, Shuntao Wang, Jinqian Mao, Chunping Liu, Wei Shi

**Affiliations:** ^1^Department of Breast and Thyroid Surgery, Union Hospital, Tongji Medical College, Huazhong University of Science and Technology, Wuhan, China; ^2^Department of Vascular Surgery, Union Hospital of Tongji Medical College, Huazhong University of Science and Technology, Wuhan, China

**Keywords:** differentiated thyroid cancer, SEER, surgery approaches, tumor size, thyroidectomy

## Abstract

**Background:**

Differentiated thyroid cancer (DTC) is the most common type of thyroid cancer. The 2015 American Thyroid Association (ATA) guidelines recommend that lobectomy is suitable for solitary intrathyroidal DTC (SI-DTC) of 1–4 cm. However, some SI-DTC patients with other high-risk characteristics still have poor prognosis and require more aggressive surgical methods. This study aimed to explore the clinical characteristics that are important for the identification and treatment of high-risk patients with SI-DTC of 1–4 cm.

**Methods:**

The study cohort was obtained from the SEER database, consisting of data between 2004 and 2013. The outcome measures were thyroid carcinoma-specific mortality (CSM) and all-cause mortality (ACM). Patient survival curves were examined using Kaplan–Meier analyses with log-rank tests and Cox proportional hazards regression analyses. Hazard ratios (HRs) were used to show the magnitude of the effect of disease stage on DTC-specific patient mortality.

**Results:**

The study included 55,947 patients with SI-DTC of 1–4 cm and 4,765 patients with DTC >4 cm. Tumor size, surgical approach, age, sex, race, and radiation exposure were independent risk factors for CSM and ACM. SI-DTC patients with female, age ≤45, and 1 cm< tumor size ≤2 cm were at low risk of CSM [HR = 0.014 (0.002–0.115)] and ACM [HR = 0.115 (0.077–0.171)] when stratified by age, sex, and tumor size. Compared to T3 patients, CSM was not significantly different in male patients, age >45, 2 cm< tumor size ≤3 cm [HR = 0.839 (0.414–1.700)] and male patients, age >45, 1 cm< tumor size ≤2 cm [HR = 0.751 (0.410–1.377)]. Furthermore, compared to T3 patients without extrathyroidal extension (ETE) and lymph node metastasis (LNM), more subgroups of SI-DTC of 1–4 cm had a similar prognosis. In addition, patients with SI-DTC of 1–4 cm showed similar rates of CSM and ACM to T3 patients without ETE, LNM, and distant metastasis (DM). Similar results were obtained when we set the age cut-off value as 55 years, according to the 8th edition of AJCC TNM system.

**Conclusions:**

Our study demonstrated that sex, age, and tumor size clearly differentiate SI-DTC of 1–4 cm into low-and high-risk categories. Survival rates were significantly lower in subgroups containing old males with larger tumors compared to younger females with small tumors. Total thyroidectomy may be favored in these high-risk subgroup patients.

## Introduction

Thyroid cancer is a frequently encountered endocrine malignancy, and differentiated thyroid cancer (DTC), namely, papillary thyroid cancer (PTC) and follicular thyroid cancer (FTC), are the most common types of thyroid cancer, accounting for about 90% of all thyroid malignancies (1–3). DTC is curable and inert with low mortality, but some patients have an aggressive disease course, such as recurrence (4, 5).

Effective risk stratification based on the evaluation of clinicopathological risk features is important for appropriate treatment of DTC patients to balance treatment, such as surgical benefits and complications (6, 7). This can be referenced in the American Thyroid Association’s guidelines (6th, 7th, and 8th versions) on the management of DTC (5, 8, 9). The 8th version (2015 American Thyroid Association (ATA) guideline) recommended that lobectomy is suitable for solitary intrathyroidal DTC (SI-DTC: without extrathyroidal invasion, lymph node metastasis, and distant metastasis) of tumor size between 1 and 4 cm and is accepted by most clinicians for the treatment of thyroid cancer.

However, the worldwide impact of this recommendation on the treatment of thyroid cancer remains controversial (10–12). Because other high-risk characteristics in patients with SI-DTC may also affect survival, this subgroup may not be suitable for union surgical strategy. Therefore, in this study, we tried to investigate the high-risk patients among the ST-DTC of 1–4 cm and attempted to provide clues for precision treatment of SI-DTC of 1–4 cm.

## Materials and Methods

### Patient Population

The DTC study cohort was obtained from the Surveillance, Epidemiology, and End Results (SEER) from 18 regions in the United States, consisting of data between 2004 and 2013. The SEER project is a United States population-based cancer registry that began in 1973 and is supported by the National Cancer Institute and the Centers for Disease Control and Prevention. It covers approximately 30% of the population of the United States and contains data across multiple geographic regions on incidence, prevalence, mortality, population-based variables, primary characteristics of the tumor, and other attributes.

### Data Collection

Study participants included patients with a diagnosis of DTC, defined by a combination of International Classification of Diseases for Oncology (ICD-O) site code C73.9 (i.e., thyroid) and diagnostic codes of papillary and/or follicular histology. The diagnostic codes used in the study included “papillary carcinoma”, “papillary adenocarcinoma”, “follicular adenocarcinoma”, and “papillary & follicular adenocarcinoma”. Patients without follow-up information were excluded from the study.

### Statistical Analyses

Patients were followed up until December 2013. The outcome measures were thyroid carcinoma-specific mortality and all-cause mortality. Patient survival curves were examined using Kaplan–Meier analyses with log-rank tests and Cox proportional hazards regression analyses. Hazard ratios (HRs) were used to show the magnitude of the effect of disease stage on DTC-specific patient mortality, and 95% confidence intervals (CIs) were used to indicate the significance of the risk. All *P*-values were 2-sided, and *P <0*.05 was considered significant. Analyses were performed using SPSS version 19.0, Stata/SE version 12 (Stata Corp), and GraphPad Prism version 6 (GraphPad Software Inc.).

## Results

### Characteristics for Patients With SI-DTC of 0–4 cm

This study assessed the prognosis of 55,947 patients with SI-DTC with tumor size 1–4 cm and 4,765 patients with DTC with tumor size >4 cm. The mean age and follow-up duration according to the different histological subtypes are shown in **Table 1**. The clinicopathological features of patients with SI-DTC of 0–4 cm were analyzed according to tumor size (**Table 1**). Age at diagnosis, sex, race, radiation, multifocality, and surgical approaches all showed significant differences among the different tumor size groups.

**Table 1:** Charectaristic for patients with EXE (−), N0, M0, tumor size 0–4 cm.

Covariate	Level	0–1 cm	1–2 cm	2–3 cm	3–4 cm	p-value
Age*		51.44 ± 13.75	48.81 ± 14.54	47.74 ± 15.43	47.77 ± 15.99	<0.001
sex	female	24,000 (83.1%)	12,643 (80.9%)	5,893 (77.7%)	2,823 (73.1%)	<0.001
	male	4,879 (16.9%)	2,979 (19.1%)	1,691 (22.3%)	1,039 (26.9%)	
race	white	24,306 (84.2%)	13,106 (83.9%)	6,219 (82.0%)	3,109 (80.5%)	<0.001
	black	2,023 (7.0%)	941 (6.0%)	588 (7.8%)	380 (9.8%)	
	other	2,550 (8.8%)	1,575 (10.1%)	777 (10.2%)	373 (9.7%)	
radiation	none of refused	22,509 (77.9%)	6,948 (44.5%)	2,820 (37.2%)	1,381 (35.8%)	<0.001
	radiation	6,370 (22.1%)	8,674 (55.5%)	4,764 (62.8%)	2,481 (64.2%)	
multifocality	no	19,763 (68.4%)	9,218 (59.0%)	4,774 (62.9%)	2,556 (66.2%)	<0.001
	yes	9,116 (31.6%)	6,404 (41.0%)	2,810 (37.1%)	1,306 (33.8%)	
surgery	lobectomy	7,306 (25.3%)	1,638(10.5%)	991 (13.1%)	579 (15.0%)	<0.001
	subtotal or near total	1,349 (4.7%)	548 (3.5%)	300 (4.0%)	169 (4.4%)	
	total	20,224 (70.0%)	13,436 (86.0%)	6,293 (83.0%)	3,114 (80.6%)	
survival months		48.71 ± 33.22	51.11 ± 33.83	52.40 ± 34.29	52.53 ± 34.29	<0.001

*Age at diagnosis.

### Risk Factors for Cancer-Specific and All-Cause Mortality

The results of the univariate Cox regression analyses demonstrated that cancer-specific mortality was associated with age, sex, race, tumor size, and treatment approaches (radiation and surgery). Furthermore, all-cause mortality was also found to be associated with age, sex, race, radiation, and surgery. Meanwhile, the multivariate Cox regression model showed that tumor size, surgical approach, age, sex, race, and radiation were independent risk factors for cancer-specific mortality and all-cause mortality (**Table 2**).

**Table 2:** Risk factors for survival: Outcome of thyroid cancer specific mortality and all-cause mortality.

Covariate	Level	Thyroid cancer specific mortality				All-cause mortality			
		Univariate cox regression		Multivariate Cox regression		Univariate Cox regression		Multivariate Cox regression	
		Hazard Ratio (95%CI)	p-value	Hazard Ratio (95%CI)	p-value	Hazard Ratio (95%CI)	p-value	Hazard Ratio (95%CI)	p-value
age at diagnosis		1.096 (1.079–1.114)	<0.001	1.099 (1.081–1.116)	<0.001	1.083 (1.079–1.087)	<0.001	1.081 (1.077–1.085)	<0.001
sex	female	ref		ref		ref		ref	
	male	2.770 (1.845–4.159)	<0.001	1.900 (1.261–2.862)	0.002	2.200 (1.994–2.427)	<0.001	1.680 (1.521–1.856)	<0.001
race	white	ref		ref		ref		Ref	
	black	0.447 (0.150–1.509)	0.208	0.570 (0.180–1.810)	0.341	1.304 (1.106–1.538)	0.002	1.467 (1.243–1.731)	<0.001
	other	1.876 (1.096–3.211)	0.022	2.130 (1.242–3.651)	0.006	0.722 (0.598–0.872)	<0.001	0.871 (0.721–1.051)	0.149
tumor size	0–1 cm	ref		ref		ref		ref	
	1–2 cm	1.476 (0.876–2.486)	0.143	1.170 (0.680–2.014)	0.571	0.897 (0.802–1.002)	0.055	1.085 (0.965–1.219)	0.172
	2–3 cm	2.966 (1.761–4.996)	<0.001	2.217 (1.279–3.843)	0.005	0.993 (0.865–1.140)	0.921	1.195 (1.035–1.380)	0.015
	3–4 cm	3.352 (1.809–6.210)	<0.001	2.171 (1.137–4.148)	0.019	1.074 (0.901–1.281)	0.426	1.160 (0.967–1.392)	0.109
multifocality	No	ref		ref		ref		ref	
	yes	1.200 (0.795–1.810)	0.386	0.886 (0.584–1.345)	0.569	0.938 (0.850–1.035)	0.205	0.982 (0.888–1.087)	0.732
radiation	none of refused	ref		ref		ref		ref	
	radiation	2.371 (1.568–3.586)	<0.001	2.185 (1.377–3.466)	<0.001	0.683 (0.620–0.753)	<0.001	0.827 (0.741–0.923)	0.001
surgery	lobectomy	ref		ref		ref		ref	
	subtotal or near total	2.118 (0.710–6.321)	0.179	1.976 (0.657–5.947)	0.226	0.904 (0.727–1.125)	0.366	1.023 (0.821–1.274)	0.838
	total	2.381 (1.197–4.735)	0.013	1.983 (0.969–4.056)	0.061	0.788 (0.705–0.880)	<0.001	0.987 (0.877–1.109)	0.822

### Subgroups Analysis With High-Risk Mortality

When three common clinical factors, age, sex, and tumor size, were included to select the high-risk mortality of SI-DTC, we found that compared to patients with male sex, age >45 years, and 3 cm< tumor size ≤4 cm, female patients with 1 cm <tumor size ≤3 cm aged ≤45 years, female patients with 0< tumor size ≤3 cm aged >45 years, and male patients with 0< tumor size ≤2 cm aged >45 years were at a lower risk of cancer-specific mortality (**Table 3**).

**Table 3:** Age, sex, tumor size factors for survival: outcome of thyroid cancer specific mortality and all-cause mortality.

Covariate	Level	Thyroid cancer specific mortality		All-cause mortality	
		Univariate Cox regression		Univariate Cox regression	
		Hazard Ratio (95%CI)	p-value	Hazard Ratio (95%CI)	p-value
0		ref		ref	
1		0.014 (0.002–0.115)	<0.001	0.115 (0.077–0.171)	<0.001
2		0.084 (0.022–0.318)	<0.001	0.098 (0.059–0.164)	<0.001
3			0.971	0.068 (0.031–0.150)	<0.001
4			0.975	0.159 (0.084–0.299)	<0.001
5			0.976	0.173 (0.092–0.326)	<0.001
6			0.981	0.217 (0.110–0.429)	<0.001
7			0.985	0.289 (0.137–0.611)	0.001
8		0.120 (0.053–0.272)	<0.001	0.593 (0.442–0.796)	0.001
9		0.217 (0.094–0.502)	<0.001	0.559 (0.411–0.760)	<0.001
10		0.342 (0.140–0.838)	0.019	0.640 (0.460–0.890)	0.008
11		0.427 (0.155–1.177)	0.1	0.817 (0.571–1.169)	0.269
12		0.275 (0.111–0.684)	0.005	1.116 (0.821–1.518)	0.484
13		0.363 (0.136–0.967)	0.043	1.137 (0.821–1.574)	0.441
14		0.910 (0.366–2.264)	0.84	1.310 (0.925–1.855)	0.128
15			0.93	0.086 (0.058–0.128)	<0.001

Ref: age >45 years old, Male, 3< tumor size ≤4 cm.

1: age ≤45 years old, Female, 1< tumor size ≤2 cm.

2: age ≤45 years old, Female, 2< tumor size ≤3 cm.

3: age ≤45 years old, Female, 3< tumor size ≤4 cm.

4: age ≤45 years old, Male, 0< tumor size ≤1 cm.

5: age ≤45 years old, Male, 1< tumor size ≤2 cm.

6: age ≤45 years old, Male, 2< tumor size ≤3 cm.

7: age ≤45 years old, Male, 3< tumor size ≤4 cm.

8: age >45 years old, Female, 0< tumor size ≤1 cm.

9: age >45 years old, Female, 1< tumor size ≤2 cm.

10: age >45 years old, Female, 2< tumor size ≤3 cm.

11: age >45 years old, Female, 3< tumor size ≤4 cm.

12: age >45 years old, Male, 0< tumor size ≤1 cm.

13: age >45 years old, Male, 1< tumor size ≤2 cm.

14: age >45 years old, Male, 2< tumor size ≤3 cm.

15: age ≤45 years old, Female, 0< tumor size ≤1 cm.

Moreover, age ≤45 years was a low-risk factor for all-cause mortality in all patients regardless of sex or tumor size when compared to male patients with age >45 years old and 3 cm< tumor size ≤4 cm. For patients aged >45 years, female sex and 0< tumor size ≤3 cm were low-risk factors for all-cause mortality (**Table 3**).

### Comparison Between SI-DTC With T3 Patients

**Figure 1:**
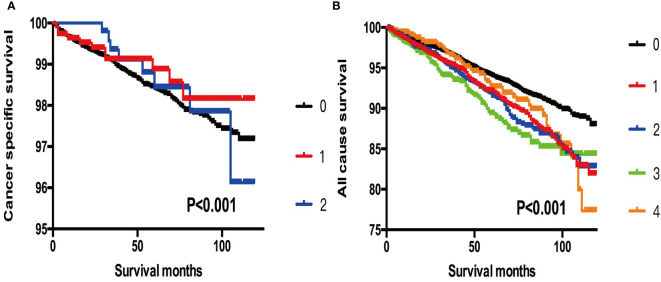
**(A)** Kaplan-Meier curves among patients stratified by tumor size for cancer-specific mortality (Log rank test, p < 0.001). 0 For patients with thyroid cancer > 4 cm in size, gross extrathyroidal extension (clinical T3), or clinically apparent metastatic disease to nodes (clinical N1) or distant sites (clinical M1). 1 Male, > 45 years old, tumor size 2-3 cm. 2 Male, > 45 years old, tumor size 3-4 cm. **(B)** Kaplan-Meier curves among patients stratified by tumor size for all-cause mortality (Log rank test, p < 0.001). 0 For patients with thyroid cancer> 4 cm, gross extrathyroidal extension (clinical T3), or clinically apparent metastatic disease to nodes (clinical N1) or distant sites (clinical M1). 1 Male, > 45 years old, tumor size 0-1 cm. 2 Male, > 45 years old, tumor size 1-2 cm. 3 Male, > 45 years old, tumor size 2-3 cm. 4 Male, > 45 years old, tumor size 3-4 cm.

When T3 patients were included for comparison, we found that the cancer-specific mortality showed no significant difference between T3 patients, SI-DTC patients with male sex, age >45 years, and 2 cm< tumor size ≤3 cm (HR: 0.839, 95%CI: 0.414–1.700), and SI-DTC patients with male sex, age >45 years, and 1 cm< tumor size ≤2 cm (HR: 0.751, 95%CI: 0.410–1.377). Similar results were obtained for all-cause mortality (**Table 4** and **Figure 1**).

**Table 4:** Analysis of si-DTC patients and patients with total T3 (ETE+, N1, M1).

Covariate	Level	Thyroid cancer specific mortality		All-cause mortality	
		Univariate Cox regression		Univariate Cox regression	
		Hazard Ratio (95%CI)	p-value	Hazard Ratio (95%CI)	p-value
0		ref		ref	
1		0.012 (0.002–0.086)	<0.001	0.150 (0.112–0.200)	<0.001
2		0.071 (0.023–0.221)	<0.001	0.128 (0.083–0.197)	<0.001
3		0		0.088 (0.042–0.186)	<0.001
4		0		0.206 (0.117–0.365)	<0.001
5		0		0.226 (0.128–0.399)	<0.001
6		0		0.284 (0.152–0.529)	<0.001
7		0		0.377 (0.188–0.756)	0.006
8		0.099 (0.063–0.157)	<0.001	0.770 (0.692–0.856)	<0.001
9		0.180 (0.110–0.295)	<0.001	0.726 (0.631–0.834)	<0.001
10		0.285 (0.160–0.510)	<0.001	0.832 (0.693–0.999)	0.049
11		0.355 (0.167–0.754)	0.007	1.063 (0.844–1.337)	0.604
12		0.228 (0.124–0.418)	<0.001	1.448 (1.260–1.664)	<0.001
13		0.301 (0.148–0.609)	0.001	1.475 (1.238–1.757)	<0.001
14		0.751 (0.410–1.377)	0.354	1.699 (1.372–2.103)	<0.001
15		0.839 (0.414–1.700)	0.627	1.311 (0.980–1.755)	0.068
16		0		0.112 (0.084–0.149)	<0.001

Ref: total T3 (includes M1, N1, ETE+).

1: age ≤45 years old, Female, 0< tumor size ≤1 cm.

2: age ≤45 years old, Female, 1< tumor size ≤2 cm.

3: age ≤45 years old, Female, 2< tumor size ≤3 cm.

4: age ≤45 years old, Female, 3< tumor size ≤4 cm.

5: age ≤45 years old, Male, 0< tumor size ≤1 cm.

6: age ≤45 years old, Male, 1< tumor size ≤2 cm.

7: age ≤45 years old, Male, 2< tumor size ≤3 cm.

8: age ≤45 years old, Male, 3< tumor size ≤4 cm.

9: age >45 years old, Female, 0< tumor size ≤1 cm.

10: age >45 years old, Female, 1 <tumor size ≤2 cm.

11: age >45 years old, Female, 2< tumor size ≤3 cm.

12: age >45 years old, Female, 3< tumor size ≤4 cm.

13: age >45 years old, Male, 0< tumor size ≤1 cm.

14: age >45 years old, Male, 1< tumor size ≤2 cm.

15: age >45 years old, Male, 2< tumor size ≤3 cm.

16: age >45 years old, Male, 3< tumor size ≤4 cm.

**Figure 2:**
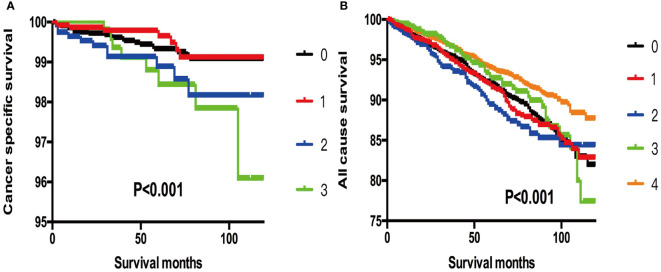
**(A)** Kaplan-Meier curves among patients stratified by tumor size for cancer-specific mortality (Log rank test, p < 0.001). 0 For patients with thyroid cancer>4 cm and without gross extrathyroidal extension (clinical T3) and no clinically apparent metastatic disease to nodes (clinical N0) or distant sites (clinical M0). 1 Male, > 45 years old, tumor size 1-2 cm. 2 Male, > 45 years old, tumor size 2-3 cm. 3 Male, > 45 years old, tumor size 3-4 cm. **(B)** Kaplan-Meier curves among patients stratified by tumor size for all-cause mortality (Log rank test, p < 0.001). 0 For patients with thyroid cancer>4 cm and without gross extrathyroidal extension (clinical T3) and no clinically apparent metastatic disease to nodes (clinical N0) or distant sites (clinical M0). 1 Male, > 45 years old, tumor size 0-1 cm. 2 Male, > 45 years old, tumor size 1-2 cm. 3 Male, > 45 years old, tumor size 2-3 cm. 4 Male, > 45 years old, tumor size 3-4 cm.

Furthermore, when we selected T3 patients with tumor size >4 cm and excluded T3 patients without ETE and LNM for reference, we found that more subgroups of SI-DTC with a tumor size of 1–4 cm had a similar prognosis (cancer-specific mortality) with T3 patients having a tumor size >4 cm. Similar results were obtained for all-cause mortality (**Table 5** and **Figure 2**).

**Table 5:** Age, T3 and sex, tumor size factors for survival: outcome of thyroid cancer specific mortality and all-cause mortality.

Covariate	Level	Thyroid cancer specific mortality			All-cause mortality		
		Univariate Cox regression			Univariate Cox regression		
		Hazard Ratio (95%CI)	p-value	Mortality (%)	Hazard Ratio (95%CI)	p-value	Mortality (%)
0		ref			ref		
1		0.019 (0.003–0.136)	<0.001	0.02	0.135 (0.099–0.184)	<0.001	0.85
2		0.110 (0.034–0.354)	<0.001	0.1	0.115 (0.074–0.180)	<0.001	0.72
3			0.967		0.080 (0.038–0.169)	<0.001	0.5
4			0.971		0.187 (0.105–0.333)	<0.001	1.05
5			0.973		0.204 (0.114–0.363)	<0.001	1.27
6			0.978		0.256 (0.136–0.481)	<0.001	1.68
7			0.983		0.340 (0.168–0.687)	0.003	2.17
8		0.154 (0.090–0.262)	<0.001	0.13	0.696 (0.603–0.805)	<0.001	3.82
9		0.279 (0.159–0.491)	<0.001	0.24	0.656 (0.553–0.778)	<0.001	3.73
10		0.442 (0.233–0.842)	0.013	0.4	0.752 (0.611–0.925)	0.007	4.5
11		0.551 (0.247–1.228)	0.145	0.5	0.960 (0.748–1.233)	0.752	5.66
12		0.353 (0.181–0.687)	0.002	0.29	1.310 (1.105–1.553)	0.002	7.07
13		0.466 (0.219–0.994)	0.048	0.39	1.334 (1.091–1.631)	0.005	7.29
14		1.166 (0.599–2.269	0.651	1	1.538 (1.216–1.945)	<0.001	8.57
15		1.301 (0.610–2.776)	0.496	1.19	1.185 (0.872–1.611)	0.279	7.15
16			0.92		0.101 (0.075–0.136)	<0.001	0.59

Ref: T3, with EXE(−), N0.

1: age ≤45 years old, Female, 0< tumor size ≤1 cm.

2: age ≤45 years old, Female, 1< tumor size ≤2 cm.

3: age ≤45 years old, Female, 2< tumor size ≤3 cm.

4: age ≤45 years old, Female, 3< tumor size ≤4 cm.

5: age ≤45 years old, Male, 0< tumor size ≤1 cm.

6: age ≤45 years old, Male, 1< tumor size ≤2 cm.

7: age ≤45 years old, Male, 2< tumor size ≤3 cm.

8: age ≤45 years old, Male, 3< tumor size ≤4 cm.

9: age >45 years old, Female, 0< tumor size ≤1 cm.

10: age >45 years old, Female, 1< tumor size ≤2 cm.

11: age >45 years old, Female, 2< tumor size ≤3 cm.

12: age >45 years old, Female, 3< tumor size ≤4 cm.

13: age >45 years old, Male, 0< tumor size ≤1 cm.

14: age >45 years old, Male, 1< tumor size ≤2 cm.

15: age >45 years old, Male, 2< tumor size ≤3 cm.

16: age >45 years old, Male, 3< tumor size ≤4 cm.

**Figure 3:**
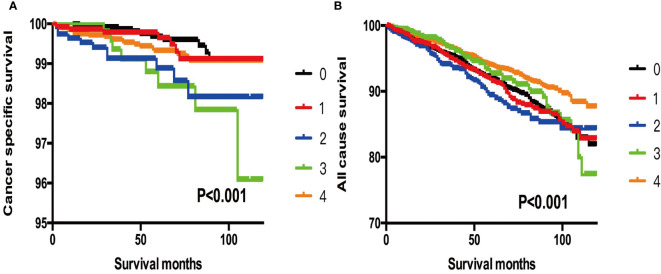
**(A)** Kaplan-Meier curves among patients stratified by tumor size for cancer-specific mortality (Log rank test, p < 0.001). 0 For patients with thyroid cancer>4 cm and without gross extrathyroidal extension (clinical T3) and no clinically apparent metastatic disease to nodes (clinical N0) and no distant sites (clinical M0). 1 Male, > 45 years old, tumor size 0-1 cm. 2 Male, > 45 years old, tumor size 1-2 cm. 3 Male, > 45 years old, tumor size 2-3 cm. 4 Male, > 45 years old, tumor size 3-4 cm. **(B)** Kaplan-Meier curves among patients stratified by tumor size for all-cause mortality (Log rank test, p < 0.001). 0 For patients with thyroid cancer>4 cm and without gross extrathyroidal extension (clinical T3) and no clinically apparent metastatic disease to nodes (clinical N0) and no distant sites (clinical M0). 1 Male, > 45 years old, tumor size 0-1 cm. 2 Male, > 45 years old, tumor size 1-2 cm. 3 Male, > 45 years old, tumor size 2-3 cm. 4 Male, > 45 years old, tumor size 3-4 cm.

In addition, when we selected T3 patients without ETE, LNM, and DM as references, we found that more subgroups and patients with SI-DTC with a tumor size of 1–4 cm showed similar rates of cancer-specific mortality and all-cause mortality (**Table 6** and **Figure 3**). Similar results were obtained when we set the age cut-off value as 55 years according to the 8th edition of the American Joint Committee on Cancer (AJCC) Tumor, Node, Metastasis (TNM) system (13) (**Tables S1–S3** and **Figures S1****–****S3**).

**Table 6:** Analysis of SI-DTC and T3 (ETE−, N0, M0) subgroup.

Covariate	Level	Thyroid cancer specific mortality		All-cause mortality	
		Univariate Cox regression		Univariate Cox regression	
		Hazard Ratio (95%CI)	p-value	Hazard Ratio (95%CI)	p-value
0		Ref		ref	
1		0.029 (0.004–0.217)	0.001	0.148 (0.108–0.201)	<0.001
2		0.172 (0.052–0.571)	0.004	0.126 (0.081–0.197)	<0.001
3				0.087 (0.041–0.185)	<0.001
4				0.204 (0.114–0.365)	<0.001
5				0.223 (0.125–0.398)	<0.001
6				0.279 (0.148–0.526)	<0.001
7				0.372 (0.184–0.752)	0.006
8		0.243 (0.135–0.437)	<0.001	0.762 (0.655–0.886)	<0.001
9		0.440 (0.238–0.816)	0.009	0.718 (0.602–0.856)	<0.001
10		0.697 (0.350–1.388)	0.305	0.822 (0.665–1.016)	0.07
11		0.868 (0.376-2.008)	0.741	1.050 (0.815–1.353)	0.705
12		0.558 (0.274–1.134)	0.107	1.433 (1.203–1.709)	<0.001
13		0.736 (0.332–1.633)	0.451	1.460 (1.188–1.793)	<0.001
14		1.844 (0.907–3.747)	0.091	1.682 (1.324–2.136)	<0.001
15		2.051 (0.925–4.548)	0.077	1.296 (0.950–1.767)	0.102
16				0.111 (0.082–0.150)	<0.001

Ref: T3, with EXE(−), N0, M0.

1: age ≤45 years old, Female, 0< tumor size ≤1 cm.

2: age ≤45 years old, Female, 1< tumor size ≤2 cm.

3: age ≤45 years old, Female, 2< tumor size ≤3 cm.

4: age ≤45 years old, Female, 3< tumor size ≤4 cm.

5: age ≤45 years old, Male, 0< tumor size ≤1 cm.

6: age ≤45 years old, Male, 1< tumor size ≤2 cm.

7: age ≤45 years old, Male, 2< tumor size ≤3 cm.

8: age ≤45 years old, Male, 3< tumor size ≤4 cm.

9: age >45 years old, Female, 0< tumor size ≤1 cm.

10: age >45 years old, Female, 1< tumor size ≤2 cm.

11: age >45 years old, Female, 2< tumor size ≤3 cm.

12: age >45 years old, Female, 3< tumor size ≤4 cm.

13: age >45 years old, Male, 0< tumor size ≤1 cm.

14: age >45 years old, Male, 1< tumor size ≤2 cm.

15: age >45 years old, Male, 2< tumor size ≤ 3cm.

16: age >45 years old, Male, 3< tumor size ≤ 4cm.

## Discussion

The current trend in the management of DTC has become more conservative. Unilateral lobectomy is now suggested as a viable alternative to total thyroidectomy for patients with SI-DTC 1–4 cm in size based on the ATA 2015 guidelines (4). Although overall prognosis seems to be excellent after lobectomy, some patients that appear to be at low risk, in fact, have high intrinsic risk for poor prognosis and should benefit from an aggressive treatment. Even in papillary thyroid microcarcinoma, cases evaluated as having a low risk of recurrence preoperatively showed intermediate to high-risk disease post-surgery, leading to a higher rate of radioiodine therapy (14).

Lobectomy for low-risk SI-DTC can be beneficial for thyroid function preservation and decreased risk of surgical complications, such as postoperative hypoparathyroidism and recurrent laryngeal nerve injury, according to many reports (15, 16). Despite these advantages, total thyroidectomy is generally accepted for SI-DTC because total thyroidectomy can provide convenience for the use of radioactive iodine in surveillance and postoperative treatment (4). It is easier to detect recurrence early *via* follow-up and dynamically monitoring serum thyroglobulin levels. In addition, Benjamin et al. reported that nearly half of low-risk DTC patients were found to have tumors in the contralateral lobe and may be more adequate for total thyroidectomy (17).

This study suggested that there were still some patients with high-risk prognosis among the SI-DTC with 1–4 cm, and selecting low-risk patients based on ETE, LNM, and DM was not precise. Our results provided some estimate for this possible need for completion of a total thyroidectomy after an initial lobectomy for these otherwise low-risk patients and can provide a future guide for the precision treatment for patients with SI-DTC of 1–4 cm.

In addition, some patients who underwent lobectomy may be reclassified as intermediate or high risk after surgery based on the histological findings, and Pedro et al. suggested that total thyroidectomy might be a better option for these patients (13). Previous retrospective studies have estimated that 40–60% of patients with low-risk 1–4 cm PTCs, if initially treated with lobectomy, would require a completion thyroidectomy due to high-risk pathological features in postoperative pathology reports (18, 19). These results underscore the importance of preoperative and intraoperative meticulous assessments by the surgeon during lobectomy for SI-PTCs.

Recently, Huang et al. (20) revealed that in tumors larger than 2.0 to 3.0 cm, recurrences of BRAF V600E mutation-positive SI-PTC were comparable with those of counterpart invasive solitary PTC and need more aggressive treatment. However, these adverse features are only apparent on histopathology and cannot be identified preoperatively. If a lobectomy has been performed, a completion thyroidectomy may be required, which may be associated with certain additional risks. It is essential to know the prevalence of these adverse pathological features to enable surgical planning and patient counseling. A recent study stated that for low-risk PTC patients, identification of intraoperative risk factors such as evidence or suspicion of invasion into local structures (namely, muscle, recurrent laryngeal nerve, esophagus, trachea, or other structures) or positive lymph nodes confirmed on intraoperative frozen section analysis can reduce the need for a later completion thyroidectomy in 21% of cases, but not exclude this need completely. Up to 30% of patients would be deemed intermediate or high risk, requiring a second operation (21). Thus, further studies are needed on preoperative risk stratification.

In this study, we included more clinicopathological factors (age, sex, and tumor size) for analysis to evaluate the reclassification of SI-DTC. Considering that age and sex are easily available preoperatively, including these characteristics for analysis may be more reasonable to select high-risk patients with SI-DTC and reconsider the treatment approaches. We found that survival rates were significantly lower in patients with male sex, age >45 years, and larger tumor size when compared to female patients with age ≤45 years and smaller tumor size, indicating that the former group have a poor prognosis and may require a more radical treatment plan. Our findings are consistent with the role of tumor size in the aggressiveness of PTC. Given that cancer-specific mortality occurred frequently in patients with 3–4 cm in size compared to other subgroups, a close preoperative and intraoperative evaluation of tumor size is important in SI-DTC patients.

A common clinical scenario for SI-DTC with 1–4 cm tumor size is that preoperative ultrasonography does not show suspicious LNM and extrathyroidal extension, as preoperative ultrasonography has a limited sensitivity in detecting central LNM; therefore, many patients with SI-DTC may have occult LNM and ETE, which can synergize with other high-risk characteristics, such as male sex and old age, in promoting DTC mortality. If such patients are treated by lobectomy without neck dissection and radioiodine ablation, the mortality risk could be higher. The present study found that the prognosis of SI-DTC was affected by other clinicopathological characteristics such as sex, age, and tumor size; thus, lobectomy for SI-DTC regardless of the specific clinical status seems to be unreasonable.

Some inherent limitations must be considered when interpreting our results. First, all the risk characteristics included clinicopathological features from the SEER database, but other factors such as ultrasonic data, molecular mutations, vascular invasion, and family history were not obtained nor included in our analysis. Second, data regarding recurrence are not captured in the SEER database, and the designation of cancer-specific death is susceptible to overestimation bias, particularly for diseases such as DTC. Furthermore, given the generally favorable prognosis of PTC, the relatively short study period and follow-up period (2010–2013) is a limitation of our analysis.

### Conclusion

Our study demonstrated that sex, age, and tumor size clearly differentiate SI-DTC with a tumor size of 1–4 cm into low and high risk categories. Survival rates were significantly lower in subgroups containing males, older patients, and patients with larger tumor size compared to females, younger patients, and patients with smaller tumor size. Therefore, total thyroidectomy may be favored for these high-risk subgroup patients, and more clinicopathological factors should be included for risk stratification in managing patients with SI-DTC with tumors that are 1–4 cm in size.

## Data Availability Statement

The raw data supporting the conclusions of this article will be made available by the authors, without undue reservation.

## Author Contributions

SW, LZ, FD, and CL designed the study. FD, LZ, WS, and JM collected and analyzed the data. FD and LZ wrote the manuscript. All authors contributed to the article and approved the submitted version.

## Conflict of Interest

The authors declare that the research was conducted in the absence of any commercial or financial relationships that could be construed as a potential conflict of interest.

The reviewer ZL declared a shared affiliation with the authors to the handling editor at time of review. The reviewer JM declared a shared affiliation with the authors to the handling editor at time of review.

## Publisher’s Note

All claims expressed in this article are solely those of the authors and do not necessarily represent those of their affiliated organizations, or those of the publisher, the editors and the reviewers. Any product that may be evaluated in this article, or claim that may be made by its manufacturer, is not guaranteed or endorsed by the publisher.
