# Supplementary material for: Clinical Characteristics-Assisted Risk Stratification for Extent of Thyroidectomy in Patients With 1–4 cm Solitary Intrathyroidal Differentiated Thyroid Cancer
Source: Front Endocrinol (Lausanne). 2022 Feb 8;12:790730. doi: 10.3389/fendo.2021.790730 (PMC8861194; doi:10.3389/fendo.2021.790730)
Supplement: Supplementary file 1 [file DataSheet_1.docx]

Table S1 Patients in SI-DTC and total T3 (ETE+, N1, M1) group were analyzed by age of 55

| covariate | level | thyroid cancer specific mortality | | all-cause mortality | |
| --- | --- | --- | --- | --- | --- |
|  |  | univariate cox regression | | univariate cox regression | |
|  |  | Hazard Ratio(95%CI) | p-value | Hazard Ratio(95%CI) | p-value |
| 0 |  | ref |  | ref |  |
| 1 |  | 0.023（0.007-0.073） | <0.001 | 0.187（0.151-0.232） | <0.001 |
| 2 |  | 0.048（0.015-0.150） | <0.001 | 0.187（0.138-0.252） | <0.001 |
| 3 |  | 0.067（0.017-0.271） | <0.001 | 0.157（0.098-0.251） | <0.001 |
| 4 |  | 0.032（0.004-0.226） | 0.001 | 0.383（0.284-0.515） | <0.001 |
| 5 |  | 0.043（0.006-0.308） | 0.002 | 0.484（0.356-0.658） | <0.001 |
| 6 |  | 0.204（0.065-0.637） | 0.006 | 0.404（0.267-0.611） | <0.001 |
| 7 |  | 0.109（0.015-0.779） | 0.027 | 0.422（0.253-0.703） | 0.001 |
| 8 |  | 0.117（0.067-0.206） | <0.001 | 1.136（1.014-1.272） | 0.028 |
| 9 |  | 0.310（0.184-0.524） | <0.001 | 1.179（1.015-1.371） | 0.032 |
| 10 |  | 0.537（0.300-0.961） | 0.036 | 1.291（1.058-1.576） | 0.012 |
| 11 |  | 0.473（0.195-1.148） | 0.098 | 1.719（1.344-2.198） | <0.001 |
| 12 |  | 0.312（0.166-0.589） | <0.001 | 1.911（1.650-2.212） | <0.001 |
| 13 |  | 0.406（0.191-0.863） | 0.019 | 1.803（1.485-2.189） | <0.001 |
| 14 |  | 0.887（0.438-1.798） | 0.74 | 2.400（1.910-3.016） | <0.001 |
| 15 |  | 1.195（0.563-2.537） | 0.643 | 1.835（1.341-2.512） | <0.001 |
| 16 |  | 0.033（0.016-0.071) | <0.001 | 0.205（0.174-0.243） | <0.001 |

Ref: total T3 (include M1, N1, ETE+)

1: age<=55 years old, Female, 0<tumor size<=1cm

2: age<=55 years old, Female, 1<tumor size<=2cm;

3: age<=55 years old, Female, 2<tumor size<=3cm;

4: age<=55 years old, Female, 3<tumor size<=4cm;

5: age<=55 years old, Male, 0<tumor size<=1cm;

6: age<=55 years old, Male, 1<tumor size<=2cm;

7: age<=55 years old, Male, 2<tumor size<=3cm;

8: age<=55 years old, Male, 3<tumor size<=4cm;

9: age>55 years old, Female, 0<tumor size<=1cm;

10: age>55 years old, Female, 1<tumor size<=2cm;

11: age>55 years old, Female, 2<tumor size<=3cm;

12: age>55 years old, Female, 3<tumor size<=4cm;

13: age>55 years old, Male, 0<tumor size<=1cm

14: age>55 years old, Male, 1<tumor size<=2cm

15: age>55 years old, Male, 2<tumor size<=3cm

16: age>55 years old, Male, 3<tumor size<=4cm

Table S2 Analysis of SI-DTC and T3 (N0, ETE-) subgroup patients by age of 55.

| covariate | level | thyroid cancer specific mortality | | all-cause mortality | |
| --- | --- | --- | --- | --- | --- |
|  |  | univariate cox regression | | univariate cox regression | |
|  |  | Hazard Ratio(95%CI) | p-value | Hazard Ratio(95%CI) | p-value |
| 0 |  | ref |  | ref |  |
| 1 |  | 0.036（0.011-0.116） | <0.001 | 0.169（0.133-0.214） | <0.001 |
| 2 |  | 0.075（0.023-0.241） | <0.001 | 0.169（0.123-0.232） | <0.001 |
| 3 |  | 0.104（0.025-0.432） | 0.002 | 0.142（0.088-0.229） | <0.001 |
| 4 |  | 0.049（0.007-0.357） | 0.003 | 0.346（0.253-0.473） | <0.001 |
| 5 |  | 0.067（0.009-0.487） | 0.008 | 0.438（0.317-0.604） | <0.001 |
| 6 |  | 0.317（0.098-1.022） | 0.054 | 0.365（0.238-0.559） | <0.001 |
| 7 |  | 0.169（0.023-1.231） | 0.079 | 0.381（0.226-0.641） | <0.001 |
| 8 |  | 0.182（0.098-0.340） | <0.001 | 1.029（0.886-1.196） | 0.706 |
| 9 |  | 0.481（0.266-0.870） | 0.015 | 1.068（0.893-1.279） | 0.471 |
| 10 |  | 0.835（0.439-1.589） | 0.583 | 1.169（0.936-1.459） | 0.169 |
| 11 |  | 0.734（0.290-1.858） | 0.514 | 1.557（1.195-2.028） | 0.001 |
| 12 |  | 0.484（0.243-0.967） | 0.04 | 1.731（1.452-2.065） | <0.001 |
| 13 |  | 0.631（0.283-1.406） | 0.26 | 1.634（1.314-2.030） | <0.001 |
| 14 |  | 1.379（0.646-2.941） | 0.406 | 2.177（1.697-2.791） | <0.001 |
| 15 |  | 1.856（0.833-4.138） | 0.13 | 1.662（1.196-2.309） | 0.002 |
| 16 |  | 0.052（0.023-0.115） | <0.001 | 0.186（0.153-0.226） | <0.001 |

Ref: T3, with EXE(-), N0

1: age<=55 years old, Female, 0<tumor size<=1cm

2: age<=55 years old, Female, 1<tumor size<=2cm;

3: age<=55 years old, Female, 2<tumor size<=3cm;

4: age<=55 years old, Female, 3<tumor size<=4cm;

5: age<=55 years old, Male, 0<tumor size<=1cm;

6: age<=55 years old, Male, 1<tumor size<=2cm;

7: age<=55 years old, Male, 2<tumor size<=3cm;

8: age<=55 years old, Male, 3<tumor size<=4cm;

9: age>55 years old, Female, 0<tumor size<=1cm;

10: age>55 years old, Female, 1<tumor size<=2cm;

11: age>55 years old, Female, 2<tumor size<=3cm;

12: age>55 years old, Female, 3<tumor size<=4cm;

13: age>55 years old, Male, 0<tumor size<=1cm

14: age>55 years old, Male, 1<tumor size<=2cm

15: age>55 years old, Male, 2<tumor size<=3cm

16: age>55 years old, Male, 3<tumor size<=4cm

Table S3 Analysis of SI-DTC and T3 (ETE-, N0, M0) subgroup patients by age of 55.

| covariate | level | thyroid cancer specific mortality | | all-cause mortality | |
| --- | --- | --- | --- | --- | --- |
|  |  | univariate cox regression | | univariate cox regression | |
|  |  | Hazard Ratio(95%CI) | p-value | Hazard Ratio(95%CI) | p-value |
| 0 |  | ref |  | ref |  |
| 1 |  | 0.057(0.017-0.188) | <0.001 | 0.185(0.145-0.235) | <0.001 |
| 2 |  | 0.117(0.035-0.389) | <0.001 | 0.185(0.134-0.254) | <0.001 |
| 3 |  | 0.164(0.039-0.694) | 0.014 | 0.155(0.096-0.251) | <0.001 |
| 4 |  | 0.078(0.011-0.573) | 0.012 | 0.379(0.276-0.519) | <0.001 |
| 5 |  | 0.106(0.014-0.779) | 0.027 | 0.479(0.346-0.663) | <0.001 |
| 6 |  | 0.498(0.150-1.650) | 0.254 | 0.399(0.260-0.612) | <0.001 |
| 7 |  | 0.266(0.036-1.963) | 0.194 | 0.416(0.247-0.701) | 0.001 |
| 8 |  | 0.288(0.147-0.563) | <0.001 | 1.126(0.963-1.317) | 0.136 |
| 9 |  | 0.760(0.401-1.442) | 0.402 | 1.169(0.972-1.406) | 0.098 |
| 10 |  | 1.318(0.662-2.624) | 0.432 | 1.278(1.019-1.603) | 0.034 |
| 11 |  | 1.160(0.444-3.029) | 0.762 | 1.703(1.302-2.227) | <0.001 |
| 12 |  | 0.766(0.368-1.595) | 0.476 | 1.895(1.580-2.272) | <0.001 |
| 13 |  | 0.997(0.431-2.306) | 0.995 | 1.787(1.432-2.231) | <0.001 |
| 14 |  | 2.181(0.984-4.837) | 0.055 | 2.382(1.851-3.066) | <0.001 |
| 15 |  | 2.931(1.268-6.777) | 0.012 | 1.818(1.305-2.533) | <0.001 |
| 16 |  | 0.081(0.035-0.188) | <0.001 | 0.203(0.167-0.248) | <0.001 |

Ref: T3, with EXE(-), N0, M0

1: age<=55 years old, Female, 0<tumor size<=1cm

2: age<=55 years old, Female, 1<tumor size<=2cm;

3: age<=55 years old, Female, 2<tumor size<=3cm;

4: age<=55 years old, Female, 3<tumor size<=4cm;

5: age<=55 years old, Male, 0<tumor size<=1cm;

6: age<=55 years old, Male, 1<tumor size<=2cm;

7: age<=55 years old, Male, 2<tumor size<=3cm;

8: age<=55 years old, Male, 3<tumor size<=4cm;

9: age>55 years old, Female, 0<tumor size<=1cm;

10: age>55 years old, Female, 1<tumor size<=2cm;

11: age>55 years old, Female, 2<tumor size<=3cm;

12: age>55 years old, Female, 3<tumor size<=4cm;

13: age>55 years old, Male, 0<tumor size<=1cm

14: age>55 years old, Male, 1<tumor size<=2cm

15: age>55 years old, Male, 2<tumor size<=3cm

16: age>55 years old, Male, 3<tumor size<=4cm
