# Supplementary material for: Clinical Characteristics-Assisted Risk Stratification for Extent of Thyroidectomy in Patients With 1–4 cm Solitary Intrathyroidal Differentiated Thyroid Cancer
Source: Front Endocrinol (Lausanne). 2022 Feb 8;12:790730. doi: 10.3389/fendo.2021.790730 (PMC8861194; doi:10.3389/fendo.2021.790730)
Supplement: Supplementary file 2 [file DataSheet_2.docx]

**Figure S1: (A)** Kaplan Meier curves among patients with age>55 and male, stratified by tumor size for cancer-specific mortality, Log rank test p < 0.001. 0 For patients with thyroid cancer>4cm, and with extrathyroidal extension(clinical T3) , or with clinically apparent lymph node metastasis(clinical N1), or distant sites (clinical M1); 1 Male, >55 years old, 0<tumor size≤1cm; 2 Male, >55 years old, 1<tumor size≤2cm,; 3 Male, >55 years old, 2<tumor size≤3cm; 4 Male, >55 years old, 3<tumor size≤4cm. **(B)** Kaplan Meier curves among patients with age>55 and male, stratified by tumor size for all cause mortality, Log rank test p < 0.001. 0 For patients with thyroid cancer>4cm, and with extrathyroidal extension(clinical T3) , or with clinically apparent lymph node metastasis(clinical N1), or distant sites (clinical M1); 1 Male, >55 years old, 0<tumor size≤1cm; 2 Male, >55 years old, 1<tumor size≤2cm; 3 Male, >55 years old, 2<tumor size≤3cm; 4 Male, >55 years old, 3<tumor size≤4cm.

**Figure S2:** (A) Kaplan Meier curves among patients wiht age>55 and male, stratified by tumor size for cancer-specific mortality Log rank test p < 0.001. 0 For patients with thyroid cancer>4cm, and without gross extrathyroidal extension(T3, ETE-), and without lymph node metastasis (N0), but include distant sites (M1); 1 Male, >55 years old, 0<tumor size≤1cm; 2 Male, >55 years old, 1<tumor size≤2cm; 3 Male, >55 years old, 2<tumor size≤3cm; 4 Male, >55 years old, 3<tumor size≤4cm. (B) Kaplan Meier curves among patients wiht age>55 and male, stratified by tumor size for all cause mortality. Log rank test p < 0.001. 0 For patients with thyroid cancer>4cm, and without gross extrathyroidal extension(T3, ETE-), and without lymph node metastasis (N0), but include distant sites (M1); 1 Male, >55 years old, 0<tumor size≤1cm; 2 Male, >55 years old, 1<tumor size≤2cm; 3 Male, >55 years old, 2<tumor size≤3cm; 4 Male, >55 years old, 3<tumor size≤4cm.

**Figure S3:** (A) Kaplan Meier curves among patients wiht age>55 and male, stratified by tumor size for cancer-specific mortality. Log rank test p < 0.001. 0 For patients with thyroid cancer>4cm, and without extrathyroidal extension(ETE-), and without lymph node metastasis (N0), and without distant sites (M0); 1 Male, >55 years old, 0<tumor size≤1cm; 2 Male, >55 years old, 1<tumor size≤2cm; 3 Male, >55 years old, 2<tumor size≤3cm; 4 Male, >55 years old, 3<tumor size≤4cm. (B) Kaplan Meier curves among patients wiht age>55 and male, stratified by tumor size for all cause mortality. Log rank test p < 0.001. 0 For patients with thyroid cancer>4cm, and without extrathyroidal extension(ETE-), and without lymph node metastasis (N0), and without distant sites (M0); 1 Male, >55 years old, 0<tumor size≤1cm; 2 Male, >55 years old, 1<tumor size≤2cm; 3 Male, >55 years old, 2<tumor size≤3cm; 4 Male, >55 years old, 3<tumor size≤4cm.
