# Supplementary figures and images for: Clinical Characteristics-Assisted Risk Stratification for Extent of Thyroidectomy in Patients With 1–4 cm Solitary Intrathyroidal Differentiated Thyroid Cancer
Source: Front Endocrinol (Lausanne). 2022 Feb 8;12:790730. doi: 10.3389/fendo.2021.790730 (PMC8861194; doi:10.3389/fendo.2021.790730)

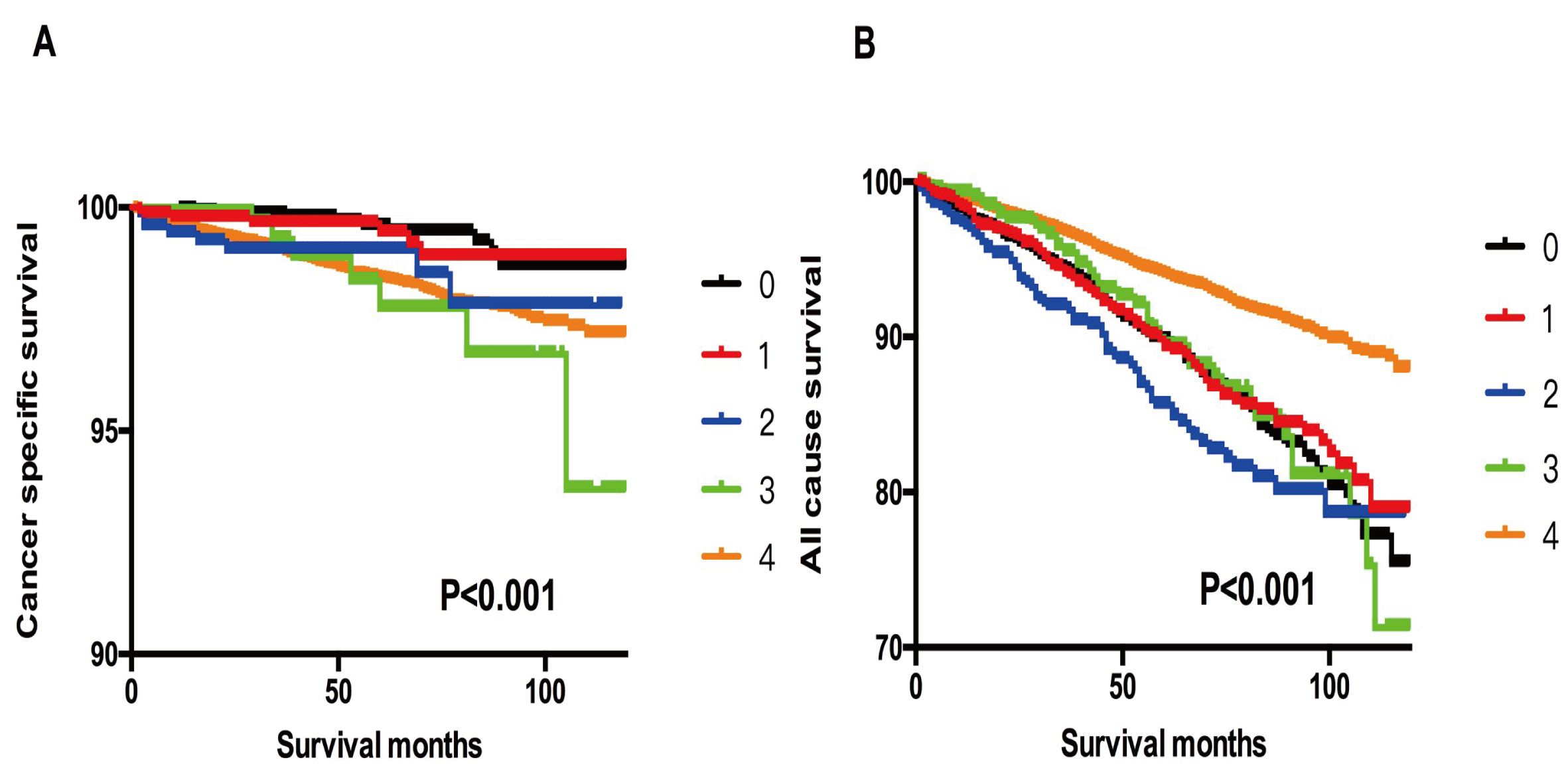

Supplement: Supplementary file 3 [file Image_1.tif]

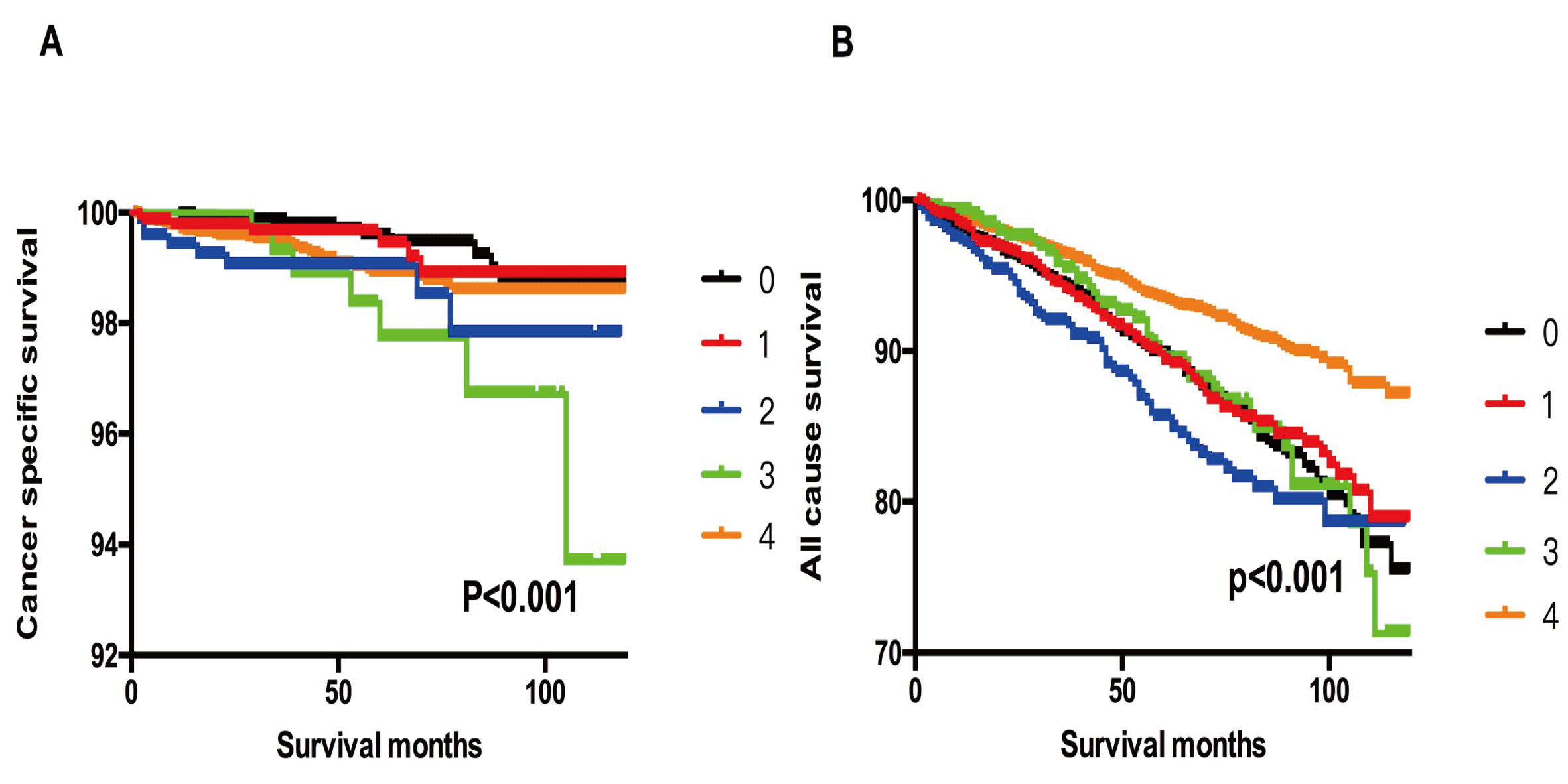

Supplement: Supplementary file 4 [file Image_2.tif]

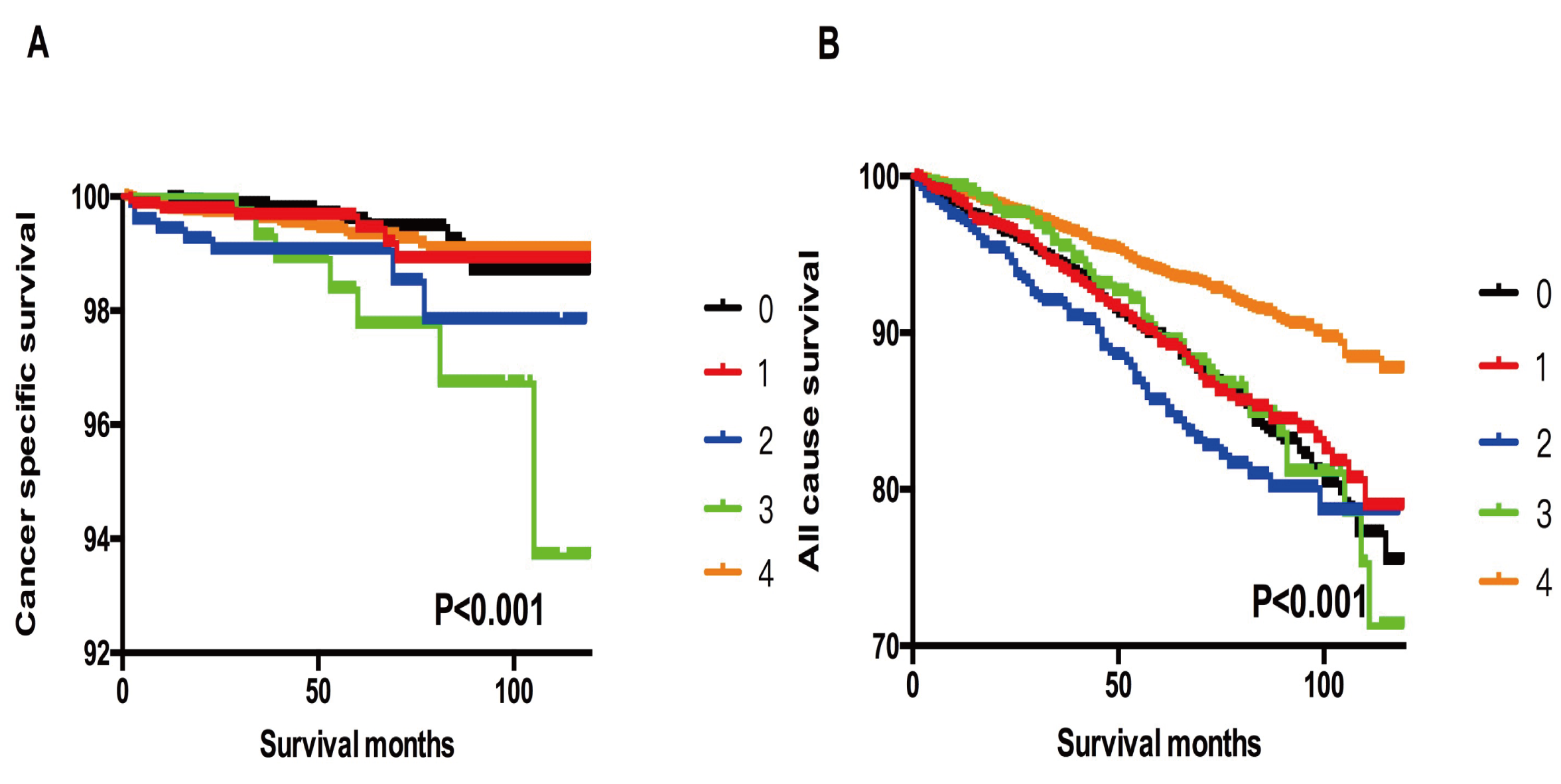

Supplement: Supplementary file 5 [file Image_3.tif]
